# Hereditary breast cancer: ever more pieces to the polygenic puzzle

**DOI:** 10.1186/1897-4287-11-12

**Published:** 2013-09-11

**Authors:** Natalia Bogdanova, Sonja Helbig, Thilo Dörk

**Affiliations:** 1 Clinics of Obstetrics and Gynaecology, Hannover Medical School, Hannover, Germany; 2 Clinics of Radiation Oncology, Hannover Medical School, Hannover, Germany

**Keywords:** Breast carcinoma, Germ-line mutations, Chromosomal instability

## Abstract

Several susceptibility genes differentially impact on the lifetime risk for breast cancer. Technological advances over the past years have enabled the detection of genetic risk factors through high-throughput screening of large breast cancer case–control series. High- to intermediate penetrance alleles have now been identified in more than 20 genes involved in DNA damage signalling and repair, and more than 70 low-penetrance loci have been discovered through recent genome-wide association studies. In addition to classical germ-line mutation and single-nucleotide polymorphism, copy number variation and somatic mosaicism have been proposed as potential predisposing mechanisms. Many of the identified loci also appear to influence breast tumour characteristics such as estrogen receptor status. In this review, we briefly summarize present knowledge about breast cancer susceptibility genes and discuss their implications for risk prediction and clinical practice.

## Introduction

Hereditary breast cancer has been formally investigated since the middle of the 19th century [1-3]. About thirty years ago, epidemiological and genetic linkage studies of multiple-case families have guided the identification of *TP53* mutations as a cause of Li-Fraumeni Syndrome [4-6] and of *BRCA1* and *BRCA2* as first genes in which mutations strongly predispose to breast and ovarian cancer [7,8]. There are further rare syndromes which include the occurrence of breast cancer as part of the disease spectrum, and the underlying genes have been identified by positional cloning. Apart from Li-Fraumeni Syndrome, these include Cowden Disease (*PTEN*) [9,10], Peutz-Jeghers Syndrome (*LKB1/STK11*) [11,12], Lynch Syndrome (*MSH2,MLH1*) [13], Bloom’s Syndrome (*BLM*) [14] and Ataxia-Telangiectasia (*ATM*) [15]. In addition, familial lobular breast cancer has been associated with germ-line mutations in *CDH1*, the gene for E-cadherin [16,17]. Although the above-mentioned syndromes are rare, they need to be kept in mind if a breast cancer patient presents with a more complex disorder or suspicious family history. For the recessive Ataxia- Telangiectasia and Bloom’s Syndrome, the monoallelic occurrence of mutations predisposes heterozygous carriers outside of syndrome families to cancer, as will be described in more detail below.

**Figure 1:**
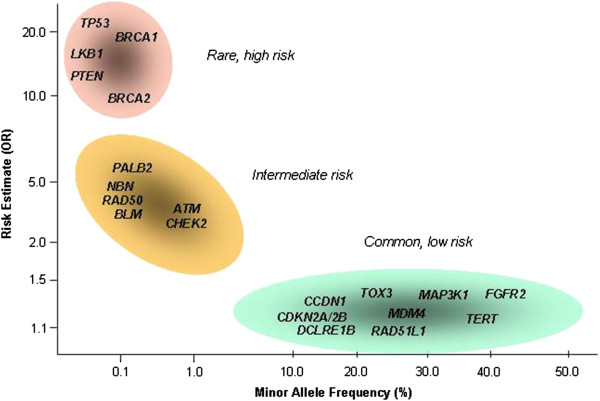
**Frequency and risk distribution of breast cancer susceptibility alleles.** Minor allele frequency of breast cancer susceptibility alleles plotted against their estimated relative risk. Selected genes are shown for high-risk, intermediate-risk and low-risk categories. Figure modified after Ref. [22].

While the identification of genes underlying these syndromes has been largely achieved through linkage analysis of large multiple-case pedigrees and positional cloning, these genes represent only a small subset of the estimated heritable fraction and further linkage studies have been unfruitful. However, hereditary breast cancer syndromes only mark the extreme end of a wide spectrum of genetically influenced breast carcinomas. During the past years evidence has been accumulated that breast cancer is a polygenic trait and also that several more susceptibility genes exist [18-21]. Their mutations have differential impact according to the minor allele frequencies and the magnitude of the allelic effect, which generally show an inversely proportional relationship (Figure 1) [22]. In the following, we briefly summarize present knowledge about breast cancer susceptibility genes and discuss their implications for risk prediction and clinical practice.

### Identification of breast cancer susceptibility alleles

The most common methods to explore the genetic basis of hereditary breast cancer have been family and linkage studies, candidate gene sequencing and case–control association studies. This has led to the identification of rare mutations conferring intermediate or high risks for breast cancer (with relative risks above two-fold) as well as multiple common polymorphic loci that harbour low-penetrance alleles.

**Figure 2:**
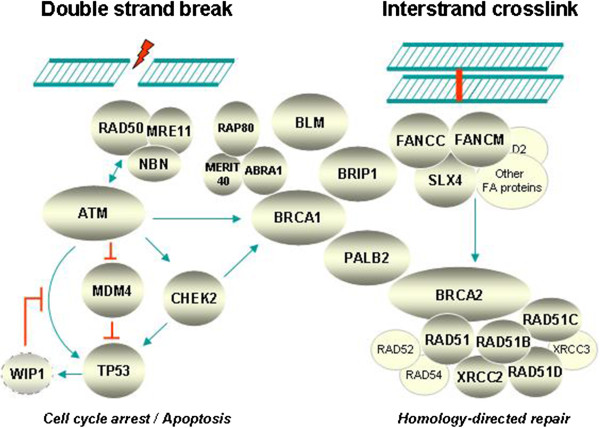
**Network of breast cancer susceptibility proteins in DNA damage signalling and repair.** Functional interplay between several known or candidate breast cancer susceptibility gene products in the intracellular response to either DNA double strand breaks (left side) or interstrand crosslinks (right side). Sensed by the Mre11-RAD50-NBN complex or by the Fanconi anemia core proteins, the respective signalling pathways merge into cell cycle arrest/apoptosis as mediated through p53, and into homology-directed recombinational repair mediated by BRCA1, PALB2, BRCA2, and the RAD51 paralogs. As mentioned in the text and in Table 1, some of the underlying genes are evidenced but have not yet been finally confirmed as *bona fide* breast cancer susceptibility genes, and some may mainly constitute ovarian cancer susceptibility genes. The genes for MERIT40, MDM4, and RAD51B harbour common polymorphisms associated with breast cancer, and *RAD51* harbours a common SNP associated with breast cancer risk in *BRCA2* mutation carriers.

I. Rare mutations with a high to intermediate penetrance:

Genes harbouring breast-cancer associated mutations with an estimated high or intermediate penetrance as summarised in Table 1. Many of them have turned out to encode proteins that act in concert with each other in an intracellular DNA damage signalling and repair network that responds to double-strand breaks or interstrand crosslinks and ensures the error-free damage removal through means of homology-directed recombinational repair (Figure 2). Nevertheless, lifetime risks can be quite different between the genes as will be described in more detail below.

**Table 1:** Genes with intermediate to high penetrance mutations for breast cancer

**Gene**	**Monoallelic mutation**	**Biallelic mutations**	**Risk for breast cancer**	**Reference**
***BRCA1***	Breast and ovarian cancer	Microcephaly and growth disorder	high	[7,23]
***BRCA2***	Breast and ovarian cancer	Fanconi anemia type D1	high	[8,24]
***TP53***	Li Fraumeni Syndrome	-	high	[5,6]
***PTEN***	PTEN harmatoma tumour syndrome (Cowden Disease)	-	high	[9,10]
***LKB1***	Peutz-Jeghers Syndrome	-	high	[11,12]
***MLH1***	Lynch Syndrome	-	probably intermediate (high for endometrial and colon cancer)	[13]
***MSH2***	Lynch Syndrome/Muir-Torre Syndrome	-	probably intermediate (high for endometrial and colon cancer)	[13]
***CDH1***	Lobular breast cancer, diffuse gastric cancer	-	high	[16,17]
***PALB2***	Breast cancer	Fanconi anemia type N	intermediate to high	[25,26]
***UIMC1***	Breast cancer^1^	-	level not yet known	[27]
***FAM175A***	Breast cancer^1^	-	level not yet known	[28]
***RAD51C***	Breast and ovarian cancer^2^	Fanconi anemia type O	low to intermediate (high for ovarian cancer)	[29,30]
***RAD51D***	Breast and ovarian cancer^2^	-	low to intermediate (high for ovarian cancer)	[31,32]
***BRIP1***	Breast and ovarian cancer	Fanconi anemia type J	low to intermediate (high for ovarian cancer)	[33,34]
***ATM***	Breast cancer, pancreatic cancer	Ataxia telangiectasia	intermediate	[15,35-39]
***MRE11A***	Breast cancer^1^	Ataxia telangiectasia-like disorder	level not yet known	[40]
***NBN***	Breast cancer, prostate cancer	Nijmegen Breakage syndrome	intermediate	[41-43]
***RAD50***	Breast cancer	Nijmegen Breakage-like disorder	intermediate	[44]
***BLM***	Breast cancer	Bloom’s Syndrome	intermediate	[45,46]
***FANCC***	Breast cancer^1^	Fanconi anemia type C	intermediate in FA blood relatives	[47,48]
***FANCM***	Breast cancer^1^	Fanconi anemia type M	probably intermediate	[49]
***SLX4***	Breast cancer^1^	Fanconi anemia type P	level not yet known	[50,51,84]
***XRCC2***	Breast cancer^1^	-	level not yet known	[52,82]
***CHEK2***	Breast cancer, prostate cancer	breast cancer	intermediate	[53-58]
***PPM1D***	Breast cancer^3^, ovarian cancer^3^	-	possibly intermediate (high for ovarian cancer), non-inherited	[59]

Legend to Table 1:

Twenty-five known or currently debated susceptibility genes harbouring intermediate or high risk mutations for breast cancer. Several of them give rise to developmental syndromes in the homozygous or compound heterozygous state as listed in the third column. The risk ranges for monoallelic mutations, as provided in column 4, are estimates for breast cancer from either family studies or case–control studies; intermediate risk 2–5, high risk > 5. ^1^Mutations in *UIMC1*, *FAM175A*, *MRE11A, FANCC, FANCM, SLX4* and *XRCC2* have been observed in very few breast cancer patients so far, therefore their possible risks are yet poorly defined. ^2^Mutations in *RAD51C* and *RAD51D* have been observed in breast cancer patients with a family history of ovarian cancer suggesting that they are primarily ovarian cancer susceptibility genes. ^3^Mutations in *PPM1D* are non-inherited, somatic mosaic mutations that have been reported to be associated with breast and ovarian cancer.

*- BRCA1* and *BRCA2*: The prototypic *BRCA1* and *BRCA2* mutations confer a very high life-time risk for breast cancer in the range of 55-85% for *BRCA1* and 35-60% for *BRCA2*, compared with an about 10% population risk [60-62]. Life-time risk for ovarian cancer is also high and may be up to 40% for *BRCA1* mutation carriers. Importantly, both the risks for breast and ovarian cancer can also be modified by additional gene loci such as SNPs in *RAD51* or *BNC2* (Refs. [63-65], and see below). The spectrum of tumours in families segregating *BRCA1* and *BRCA2* mutations includes pancreatic, prostate, colon and skin cancers. Monoallelic *BRCA2* mutations have also been associated with male breast cancer and have been observed in Li-Fraumeni families. Biallelic mutations in *BRCA2* give rise to the recessive developmental disorder, Fanconi Anemia D1 [23]. In case of *BRCA1*, homozygosity for severe mutations has not been confirmed and may be embryonically lethal. However, compound heterozygosity for two *BRCA1* mutations, one of them apparently hypomorphic, has been described in a single patient with short stature, microcephaly and early ovarian cancer [66]. Consistent with these findings, the *BRCA1* and *BRCA2* genes both encode proteins involved in the repair of DNA double strand breaks [67]. While BRCA2 is mainly involved in homology-directed recombinational repair, BRCA1 may serve as a regulatory platform more upstream in assisting the signalling of breaks and the choice of repair pathways. BRCA1 is also involved in the transcriptional regulation of the estrogen and progesterone receptors. *BRCA1* mutated breast cancers are usually estrogen-receptor negative and have a basal phenotype [67], while *BRCA2* mutated tumours exhibit a broader spectrum of phenotypes.

*BRCA1* and *BRCA2* mutations are usually truncating, although pathogenic missense mutations have also been described in crucial functional domains such as the *BRCA1* RING domain. There seems to be allele-specific expressivity as some of the mutations appear to confer higher risks for ovarian cancer than others, and ovarian cluster regions have been defined for both genes [68-70]. It has also been noted that not all mutations in *BRCA1* and *BRCA2* are highly penetrant for breast or ovarian cancer. Variants such as p.R1699Q in *BRCA1* or p.K3326X in *BRCA2* seem to be associated with rather low, though significant, breast cancer risks [71,72]. This indicates that, although *BRCA1* and *BRCA2* are often referred to as “high-penetrance genes”, their mutational heterogeneity may produce a more diverse spectrum of allelic effects.

*- PALB2*: Subsequently, the “partner and localiser of BRCA2”, *PALB2,* has been identified as another breast cancer susceptibility gene [73,74]. The PALB2 protein bridges BRCA1 and BRCA2 and synergizes in their function in recombinational DNA repair. Mutations in *PALB2* predispose to breast cancer and gastric cancer, and the penetrance for breast cancer in Finnish multiple-case families has been found similarly high as for *BRCA2* mutations [25]. There is less evidence that *PALB2* mutations predispose to ovarian cancer, although founder mutations have been identified in ovarian cancer patients from Poland and Russia [75,76]. Another founder mutation in *PALB2* is recurrent in British and Australian breast cancer patients, including multiple-case families [26]. Altogether, *PALB2* emerges as a third important breast cancer susceptibility gene with moderate- to high penetrance mutations for breast cancer.

- *UIMC1*/ *FAM175A/ BABAM1*: The binding of BRCA1 to ubiquitylated and sumoylated histones at the site of double strand breaks is mediated by the ubiquitin-interaction motif containing protein UIMC1 (better known as RAP80) through binding the *FAM175A* gene product ABRAXAS (also known as ABRA1 or CCDC98) that interacts with BRCA1 in a complex stabilised by MERIT40, the product of the *BABAM1* gene [77]. Only few studies have addressed the role of *UIMC1* or *FAM175A* mutations in breast cancer susceptibility. Familial breast cancer screening has revealed a rare alteration in the RAP80 UIM domain that impairs DNA damage response function [27], and an ABRAXAS mutation that disrupts nuclear localisation has been observed in breast cancer patients with mainly lobular tumour histology [28]. In addition, *BABAM1* has emerged as a significant low-penetrance risk locus for triple-negative breast cancer in genome-wide association studies as will be discussed further below.

*- RAD51 paralogs*: The fact that BRCA1, BRCA2 and PALB2 function together in the homology-directed recombinational repair of DNA double-strand breaks has soon prompted further investigation of candidate genes in this biological pathway. RAD51 is a key protein that mediates homologous recombination but apart from rare missense variants with uncertain significance, there have been no clearly pathogenic mutations in the coding region of the *RAD51* proto-oncogene [78]. However, a regulatory variant 135G/C in the *RAD51* promoter acts as a genetic modifier of *BRCA2* mutations [63]. Similarly, low-penetrance variants at the *RAD51L1* locus (also known as *RAD51B*) have recently been associated with breast cancer (see further below). Mutation analyses in further genes of RAD51 paralogs have uncovered *RAD51C* and *RAD51D* as susceptibility genes in hereditary breast and ovarian cancer families [29-31]. The initial data indicated that these mutations were specifically associated with a family history of ovarian cancer and were not overrepresented in breast cancer patients outside of ovarian cancer families [29-32]. However, mutations in *RAD51C* and *RAD51D* are collectively very rare and their risk pattern and tumour spectrum remains to be fully explored. Additional components of homologous recombinational repair complexes include RAD52, RAD54, XRCC2 and XRCC3. The *RAD52* gene harbours two polymorphic stop codons which did not appear to confer a largely increased breast cancer risk, although minor risks have not been excluded [79,80]. A single missense variant but no clearly pathogenic mutation has been reported in *RAD54*[81]. A potentially disease-causing mutation has been found in *XRCC2* in a recent exome sequencing study of British breast cancer patients [82]. However, follow-up studies in other European populations did not detect *XRCC2* mutations indicating that these are very rare [52]. Altogether, mutations in RAD51 paralogs appear to exist at a low level in breast cancer but their contribution is small in most, if not all populations.

*- Additional Fanconi Anemia genes*: Homology-directed recombinational repair complexes are a conserved repair platform that are shared by at least two convergent signalling pathways, the ATM-mediated signalling pathway triggered by DNA double-strand breaks, and the Fanconi Anemia protein pathway triggered by interstrand crosslinks. Since it had been found that some breast and ovarian cancer susceptibility alleles, e.g. in *BRCA2* (the *FANCD1* gene), *PALB2* (the *FANCN* gene) or *RAD51C* (the *FANCO* gene), cause Fanconi Anemia (FA) in the homozygous state, it has been reasonable to assess further FA genes for their role in breast and ovarian cancer. So far, mutations of the *BRIP1* gene have been associated with FA in the biallelic state and with breast cancer in the monoallelic state, although the risk for breast cancer appears moderate [33]. The BRIP1 protein, also known as FANCJ or BACH1, acts as a BRCA1- associated helicase, and mutations of *BRIP1* also predispose to ovarian cancer with apparently higher penetrance [34]. There is less evidence implicating the FA core proteins in breast cancer [83] but exome sequencing did uncover truncating mutations of *FANCC* and of *FANCM* in single studies [47,49] and rare truncating mutations have also been observed in *SLX4* (the *FANCP* gene) [50,51,84] suggesting that more FA genes might harbour rare breast cancer susceptibility alleles at a very low frequency. Their penetrance is unknown, however, the difficulties to confirm very early reports of an increased breast cancer risk in obligate heterozygotes from FA families may indicate that the risks are genetically heterogeneous and moderate on average, with a possible preponderance of *FANCC*[48].

*- ATM*: It has been known for long that blood relatives of patients with the neurodegenerative disorder Ataxia-Telangiectasia (A-T) face an increased breast cancer risk [35]. Homozygous A-T patients usually do not survive into late adulthood, but a few females with attenuated A-T have been reported to develop bilateral breast cancer [15]. The gene mutated in Ataxia-Telangiectasia, *ATM*, encodes a master protein kinase that orchestrates the cellular response to DNA double-strand breaks and controls via phosphorylation hundreds of proteins involved in cell cycle control, repair and apoptosis, among them BRCA1, BRCA2, BLM, TP53, CHEK2 and many other tumour suppressors [36]. Truncating mutations in *ATM* appear to confer an about three-fold increased breast cancer risk to heterozygous carriers [37-39], and heterozygotes may account for 0 · 5-1% of most populations.

*- MRE11A/ RAD50/ NBN*: At the early steps of DNA double strand break signalling, chromosome breaks are sensed and the ATM protein is activated via the MRN complex consisting of the proteins MRE11A, RAD50, and NBN [85]. The *NBN* gene underlies Nijmegen Breakage Syndrome (NBS), which is most prevalent in Eastern Europe due to a Slavic founder mutation [86]. While biallelic mutations cause NBS, a cancer-prone developmental condition with early mortality, heterozygous carriers face an about 3–5 fold increased breast cancer risk [41-43]. Similarly, biallelic mutations in *RAD50* give rise to a NBS-like disorder whereas heterozygotes for a Finnish founder mutation are predisposed towards breast cancer [44,87]. *MRE11A* also is a gene for an A-T like disorder though there has been only one study to associate *MRE11A* mutations with breast cancer so far [40,88]. Germ-line mutations in either of the three genes were also identified in an ovarian cancer sequencing study [89]. Thus, similar to the Fanconi anemia proteins, several components of DNA double strand break sensing complexes seem to be target of germ-line mutations in breast and ovarian cancer susceptibility.

- *BLM*: Another such gene that has recently been implicated in breast cancer susceptibility, is *BLM*, the gene mutated in Bloom’s Syndrome [90]. Bloom’s Syndrome (BS) is an autosomal recessive syndrome associated with short stature, premature aging and a high propensity to develop malignancies including breast cancer [14]. Cells from BS patients exhibit enhanced levels of sister chromatid exchanges, which reflects a hyperrecombinational phenotype as a consequence of *BLM* mutations and dysfunction of the encoded RecQ-type DNA helicase. A nonsense mutation in *BLM*, initially been observed in few BS patients, has been associated with breast cancer in Slavic populations, and the presently available evidence for BS mutations indicates an approximately 2–5 fold increase in breast cancer risk for heterozygotes [45,46,91].

*- CHEK2*: One of the major targets of the ATM kinase is CHEK2 which itself phosphorylates further tumour suppressor proteins, including p53 and BRCA1, in response to DNA damage [92]. *CHEK2* had initially been found mutated in Li-Fraumeni patients and one of these mutations, c.1100delC, has subsequently been associated with familial breast cancer [53,54]. Heterozygous carriers have been reported with a 2–3 fold increase in breast cancer risk, with rare homozygotes being found at a much higher risk [55,56]. In Eastern Europe, two further truncating mutations have been associated with at least similarly high breast cancer risks, whereas a missense mutation, p.I157T, has a lower penetrance [41,57,58]. There has also been some evidence for an association of *CHEK2* mutations with ovarian cancer and for additional malignancies suggesting a more general role in cancer predisposition [89,93]. It is interesting to note that, although CHEK2 interacts with BRCA1 in the same pathway, its mutations are significantly associated with estrogen receptor positive breast tumours, indicating an impact on tumour etiology that is different from BRCA1.

*- PPM1D*: Large-scale sequencing has identified truncating mutations in the p53-inducible protein phosphatase *PPM1D* which were specifically associated with breast cancer and ovarian cancer [59]. *PPM1D* encodes the oncogenic phosphatase WIP1 that antagonizes ATM-mediated p53 phosphorylation. By contrast with the other genes discussed above, all of the identified *PPM1D* mutations were mosaic in lymphocyte DNA and, where tested, were not observed in breast or ovarian tissue, suggesting a late origin in the germ-line. Their mechanism of action in breast or ovarian cancer development is presently unknown. Somatic mosaicism has previously been observed for *TP53* mutations outside of Li-Fraumeni families [89] suggesting that, in addition to classical heritable genetic factors, mosaic mutations may also contribute to the genetic predisposition to breast and ovarian cancer. These observations, if confirmed, could have important consequences for mutational screening as well as counselling. Certainly, the origin and frequency of mosaic mutations need to be studied in more detail before final conclusions can be derived.

II. Polymorphic variants with low penetrance:

Beyond the genes with relatively rare mutations discussed above, common polymorphisms have been predicted to significantly impact on risk and prevention if breast cancer were regarded as a polygenic disease [94]. Several polymorphic loci are meanwhile known which influence the risk of breast cancer (Table 2). This has been mainly achieved through genome-wide association studies (GWAS) of single nucleotide polymorphism by large consortia during the past six years. The published GWAS efforts have uncovered over 70 genomic loci for breast cancer at a genome-wide significance level [72,95-118]. All these loci harbour low-penetrance alleles with allelic odds ratios less than 1 · 5. Apart from a coding variant in *DCLRE1B* (the gene for the SNM1B/Apollo protein involved in DNA cross-link repair) and synonymous variants in *BABAM1* and *TERT,* the majority of identified variants are either intronic or intergenic. The observed intronic and intergenic variants may affect genomic regions important for the regulation of gene expression and/or gene function. As these loci still explain only a small part of the heritable fraction, it is likely that the numbers will increase rapidly. Presently known GWAS loci now cover approximately 15% of the familial relative risk, compared to about 21% captured by moderate- to high penetrance alleles. But evidence suggests that several hundreds of low-penetrance breast cancer loci might exist, meaning that even with the numbers reached so far, studies have merely grazed the surface of the iceberg [72].

**Table 2:** Genomic loci harbouring low-penetrance breast cancer susceptibility alleles

**Locus**	**SNP**	**Reported gene**	**Nearby genes (selected)**	**Association with ER status**	**Reference**
1p11	rs2580520, rs11249433	*EMBP1*	*HIST3, HIST2H2BA*	ER + ve ~ ER-ve	[99,101,119,120]
1p13	rs11552449	*DCLRE1B*	*PTPN22, HIPK1, BCL2L15*	ER + ve > ER-ve	[72]
1p36	rs616488	*PEX14*	*KIF1B, UBE4B, RBP7*	ER-ve > ER + ve	[37]
1q32	rs4245739	*MDM4*	*PIK3C2B*	ER-ve	[115]
1q32	rs6678914	*LGR6*	*UBE2T, PTPN7*	ER-ve	[115]
2p24	rs12710696	intergenic	*OSR1*	ER-ve	[115]
2q14	rs4849887	intergenic	*INHBB, RALB, GLI2*	ER + ve ~ ER-ve	[72]
2q31	rs2016394	intergenic	*DLX1, DLX2, ITGA6, PDK1*	ER + ve	[72]
2q31	rs1550623	intergenic	*CDCA7, MLK7-AS1, ZAK*	ER + ve ~ ER-ve	[72]
2q33	rs1045485, rs10931936, rs3834129- rs6723097-rs3817578	*CASP8*	*CASP10, ORC2, CDK15*	ER + ve ~ ER-ve	[101,121,122]
2q34	rs13393577	*ERBB4*	*MIR4776*	ER + ve ~ ER-ve	[110]
2q35	rs13387042, rs16857609	*DIRC3*	*PINC, TNS1, IGFBP1, IGFBP5*	ER + ve > ER-ve	[72,97,103,123,124]
3p24	rs4973768	*SLC4A7*	*NEK10*	ER + ve > ER-ve	[98,101,103]
3p24	rs12493607	*TGFBR2*	*GADL1*	ER + ve	[72]
3p26	rs10510333	intergenic	*GRM7*	ER + ve ~ ER-ve	[113]
3p26	rs6762644	*ITPR1*	*SUMF1, BHLHE40*	ER + ve	[72]
3q25	rs6788895	*SIAH2*	*MED12L, SELT, EIF2A*	ER + ve	[112]
3q26	rs3806685	intergenic	*PIK3CA, ZNF639*	ER + ve ~ ER-ve	[110]
4q24	rs9790517	*TET2*	*PPA2*	ER + ve	[72]
4q34	rs6828523	*ADAM29*	*GLRA3*	ER + ve	[72]
5q11	rs889312, rs16886165	intergenic	*MAP3K1, MIER3*	ER + ve > ER-ve	[95,99,101]
5q11	rs1353747, rs10472076	*PDE4D*	*RAB3C, PDK2*	ER + ve ~ ER-ve	[72]
5p12	rs4415084, rs10941679, rs7716600, rs9790879, rs4866929	intergenic	*HCN1, MRPS30,FGF10*	ER + ve > ER-ve	[96,97,101,103,124]
5p15	rs1092913	intergenic	*MARCH6, DAP*	ER + ve > ER-ve	[105,110]
5p15	rs2736108, rs10069690, rs2242652	*TERT*	*CLPTM1L*	variant specific	[104,114]
5q33	rs1432679	*EBF1*	*RNF145, UBLCP1*	ER + ve ~ ER-ve	[72]
6p23	rs204247	intergenic	*RANBP9, SIRT5, CCDC90A*	ER + ve	[72]
6p24	rs9348512	intergenic	*GCNT2, PAK1IP1, TFAP2A*	*BRCA2* specific	[118]
6p25	rs11242675	intergenic	*FOXQ1, FOXF2, FOXC1*	ER + ve ~ ER-ve	[72]
6q14	rs17529111, rs17530068	intergenic	*FAM46A, IBTK, SSBP2*	ER + ve ~ ER-ve	[72,109]
6q25	rs9498283	*TAB2*	*SUMO4, LATS1*	ER + ve ~ ER-ve	[110]
6q25	rs3757318, rs12662670, rs6929137, rs3734804, rs3734805, rs2046210	intergenic	*ESR1*	variant specific	[100,101,103,106,125-127]
7q32	rs2048672	*FLJ43663*	*MIR29A, KLF14*	not mentioned	[106]
7q35	rs720475	*ARHGEF5*	*NOBOX*	ER + ve	[72]
8p12	rs9693444	intergenic	*DUSP4, KIF13B*	ER + ve ~ ER-ve	[72]
8q21	rs6472903, rs2943559	*HNF4G*	*CRISPLD1, ZFHX4*	ER + ve > ER-ve	[72]
8q24	rs672888, rs1562430, rs13281615, rs11780156	intergenic	*PVT1, MIR1204-08, MYC*	ER + ve ~ ER-ve	[72,95,101,103]
9p21	rs1011970	*CDKN2B*	*CDKN2A, CDKN2B-AS1*	ER + ve > ER-ve	[101]
9q31	rs865686, rs10759243	intergenic	*RAD23B, KLF4*	ER + ve > ER-ve	[72,103]
10p12	rs7072776, rs11814448	intergenic	*DNAJC1, MLLT10*	ER + ve ≠ ER-ve	[72]
10p15	rs2380205	intergenic	*ANKRD16, FBXO18, GDI2*	ER + ve ~ ER-ve	[101]
10q21	rs10822013, rs10995190	*ZNF365*	*EGR2, NRBF2*	ER + ve > ER-ve	[101,106]
10q22	rs704010, rs12355688	*ZMIZ1*	*PPIF, ZCCHC24, EIF5AL1*	ER + ve ~ ER-ve	[101,113]
10q25	rs7904519	*TCF7L2*	*ZDHHC6, CASP7, DCLRE1A*	ER + ve ~ ER-ve	[72]
10q26	rs2981582, rs11199914, rs2981579, rs1219648, rs10510102	*FGFR2*	*WDR11, TACC2*	ER + ve	[72,95,96,99,101,103,124]
11p15	rs3817198, rs909116	*LSP1-TNNT3*	*CTSD, DUSP8, IGF2*	ER + ve ~ ER-ve	[95,101,128]
11q13	rs3903072	intergenic	*RELA, MAP3K11, MUS81*	ER + ve	[72]
11q13	rs614367, rs661204, rs78540526, rs554219, rs657686, rs75915166	intergenic	*CCND1, FGF19, FGF4, FGF3*	ER + ve > ER-ve	[101,116]
11q24	rs11820646	intergenic	*BARX2, NFRKB, PRDM10*	ER + ve ~ ER-ve	[72]
12p11	rs10771399	*PTHLH*	*CCDC81*	ER + ve ~ ER-ve	[108]
12p13	rs12422552	intergenic	*ATF7IP, GRIN2B, PLBD1*	ER + ve ~ ER-ve	[72]
12q22	rs17356907	intergenic	*NTN4, USP44, METAP2, NR2C1 VEZT, FGD6, CCDC38*	ER + ve ~ ER-ve	[72]
12q24	rs1292011	intergenic	*TBX3*	ER + ve > ER-ve	[108]
14q13	rs2236007	*PAX9*	*NKX2-8*	ER + ve > ER-ve	[72]
14q24	rs999737, rs2588809, rs1314913, rs10483813, rs8009944,	*RAD51B*	*ZFP36, ACTN1, DCAF5*	ER + ve	[72,99,101,111,119]
14q31	rs4322600	*GALC*	*GPR65*	ER + ve ~ ER-ve	[113]
14q32	rs941764	*CCDC88C*	*GPR68, SNORA11B, RPS6KA5, SMEK1, CATSPERB, TC2N*	ER + ve	[72]
16q12	rs3803662	intergenic	*TNRC9/TOX3, MIR548, CHD9*	ER + ve > ER-ve	[95,101,103,107,111,124]
16q12	rs17817449, rs11075995	*FTO*	*AKTIP, RBL2, CHD9*	ER + ve ~ ER-ve	[72,115]
16q23	rs13329835	*CDYL2*	*CENPN, ATMIN, GCSH, PKD1L2*	ER + ve > ER-ve	[72]
17q22	rs6504950, rs1156287	*STXBP4*	*COX11, HLF*	ER + ve > ER-ve	[98,101]
17q24	rs11077488	intergenic	*KCNJ2, KCNJ16*	Not mentioned	[110]
18q11	rs527616, rs1436904	*CHST9*	*KCTD1, TAF4B*	ER + ve > ER-ve	[72]
19p13	rs8170, rs8100241, rs2363956	*BABAM1*	*ANKLE1*	ER-ve	[102,109,129]
19p13	rs4808801	*ELL*	*SSBP4, FKBP8, PDE4C*	ER + ve ~ ER-ve	[72]
19q13	rs3760982	intergenic	*ZNF Cluster, KCNN4, SMG9, XRCC1*	ER + ve ~ ER-ve	[72]
19q13	rs10411161, rs3848562	*ZNF577*	*MIR125A, ZNF Cluster*	Not mentioned	[105]
20q11	rs2284378	*RALY*	*ASIP, EIF2S2, CHMP4B, ZNF341, E2F1*	ER-ve	[109]
21q21	rs2823093	intergenic	*NRIP1*	ER + ve > ER-ve	[108]
22q12	rs132390	*EMID1*	*KREMEN1, CHEK2, EWSR1, NF2*	ER + ve ~ ER-ve	[72]
22q13	rs6001930	*MKL1*	*SGSM3, ADSL, MCHR1, XPNPEP3, DNAJB7, RBX1*	ER + ve ~ ER-ve	[72]
22q13	CNV2576, tagged by rs12628403	*APOBEC3A- APOBEC3B*	*APOBEC3C*	ER + ve ~ ER-ve	[130]

Legend to Table 2:

72 genomic loci that have been found to harbour low-penetrance breast cancer susceptibility alleles. Genes already mentioned in Table 1 have been excluded although long-range effects remain a possibility. All loci except for *CASP8* have been derived from genome-wide association studies. Some chromosomal loci that harbour more than one independent risk variant were here combined when there was a strong overlap of candidate genes. If the variant was within a gene, this is listed separately, although this does not necessarily mean it represents the causal gene. Selected candidate genes in the vicinity (< 1 Mb) are listed in the fourth column. Genes were taken from the GRCh37.p10 primary assembly drawn from the NCBI Genbank (http://www.ncbi.nlm.nih.gov/gene). Association with ER status has been drawn from the original references, and a preponderance of one subtype was assumed if p(het) < 0.05. Note that genome-wide significance has been borderline for some results [106,110,113] so that additional validation may be needed for those variants.

Many of the identified GWAS loci appear to be specific for breast carcinomas. For example, the gene for fibroblast growth factor receptor 2, *FGFR2*, harbours variants associated with breast but not ovarian cancer [95,96] and breast cancer-associated variants in this gene appear to regulate the transcriptional activation of *FGFR2* in an estrogen-dependent manner [131]. The interaction with estrogen signalling may also explain why the association of some variants is restricted to ER-positive breast carcinomas (Table 2). Several of the GWAS loci further modify the risk for *BRCA1* or *BRCA2* mutation carriers [132]. In some instances, variants have been observed to differentially associate with breast cancer risk in *BRCA1* or *BRCA2* carriers, and one variant has been reported to specifically associate with *BRCA2* mutations [118]. Additionally, variants at the *RAD51L1* and *TOX3* loci have independently been identified in a GWAS for male breast cancer [111].

A minor group of common susceptibility loci has turned out to be relevant for other common cancers as well, perhaps due to their general relevance for genome integrity [133]. Some loci appear to influence both breast and ovarian cancer risk such as *BABAM1, TERT*, and the protooncogene *MYC* on chromosome 8q24. Variants at the *BABAM1* locus, encoding a BRCA1 binding partner also known as MERIT40, have been specifically associated with triple-negative breast cancer and serous epithelial ovarian cancer, which resembles the picture seen with *BRCA1* mutations [102,134]. A closer inspection of the *TERT* locus, encoding a component of telomerase, has uncovered three independent regions of strong association with breast or ovarian cancer that only partially overlap and appear to act through different mechanisms of transcriptional regulation or splicing, respectively [114]. Similarly, a closer inspection of the 8q24 locus upstream of *MYC* has indicated that the associations with different cancers were caused by independent variants at the same locus, possibly explained by tissue-specific regulation of gene expression through long-distance effects of enhancer regions [135]. These findings illustrate that, in several instances, low-penetrance breast cancer susceptibility alleles may exert regulatory roles in the fine-tuning of gene expression in the respective tissue, and the patterns of regulation can be complex.

As a caveat, a GWAS roughly localises but usually does not yet identify the causal variant. In several cases there is more than one candidate gene in the region spanned by the associated LD block, and there can be even more candidate genes under putative regulatory control of the identified locus. For example at the 5q11.2 locus, the *MAP3K1* gene represents an excellent candidate as it represents one of the most frequently mutated genes in breast tumours but *MIER3* is another mammary tumour suppressor gene nearby [136,137]. In some instances, available microarray data supported an association of the identified SNP with gene expression [72,138]. One locus, *LSP1*, lies in proximity to the imprinted region *H19/IGF2*, and breast cancer risk has been reported to be limited to the paternally inherited allele [128]. In other instances, identified loci have independently been correlated with previously known risk factors for breast cancer, such as *FTO* for obesity, *INHBB* for breast size or *ZNF365* for mammographic density, strongly suggesting that the risk for breast cancer could be mediated via these physiological traits [139-142]. But for the majority of loci, fine-mapping approaches in different ethnic populations as well as gene expression and chromatin configuration studies are presently being used to further trace down the true predisposing variants. A combination of such approaches has recently identified regulatory mechanisms that underlie the association of independent variants at 11q13 with breast cancer and act in concert to orchestrate cyclin D1 expression [116].

Copy number variants (CNVs) have also been investigated at a genome-wide level. While one GWAS did not detect a significant association for breast cancer in European patients another one detected a significant association with a common *APOBEC3B* deletion in Chinese breast cancer patients [130,143]. *APOBEC3B* encodes a cytosine deaminase that functions in localised hypermutation (“kataegis”) and may be responsible for chronic DNA damage in breast cancers [144,145]. Loss of one or both *APOBEC3B* copies was associated with odds ratios of 1 · 31 and 1 · 76, respectively [130]. Additional recent studies also showed a consistent increase in the frequency of rare CNVs in breast cancer cases when compared to controls [146,147], with a particular enrichment of CNVs in genes involved in estrogen signalling and DNA double strand break repair in one study [147]. If confirmed, this mirrors some results from genome-wide SNP analyses, although there has been no overlap of the identified loci thus far.

### Implications for risk prediction and therapy

Hereditary breast cancer represents a challenge in terms of genetic counselling as well as preventive and therapeutic decisions. The identification of mutations in individuals from multiple-case families with breast cancer makes it possible to predict the age-dependent risk for different cancers, including recurrence risks in the already affected, and to counsel patient and blood relatives more appropriately. With the identification of modifying variants, the risk prediction for *BRCA1* or *BRCA2* mutation carriers can be further refined. Risk prediction may lead to an increased surveillance or targeted prevention including magnetic resonance imaging, medication (such as tamoxifen) or preventive surgery (such as prophylactic oophorectomy). In many countries, this is available to patients with a high (over 30%) lifetime risk such as *BRCA1* or *BRCA2* mutation carriers, whereas a more restrained position is taken for patients with intermediate-penetrance mutations conferring an about 3-fold increase in breast cancer risk such as *ATM* or *CHEK2*. Although the female carriers for those mutations could also benefit from increased surveillance, large studies on the efficacy of such measures are lacking. No further counselling is provided for patients carrying common risk alleles at polymorphic loci, as these risks are too small individually to be clinically meaningful. This situation may change, however, if one considers cumulative effects for several of those variants that can reach substantial risk modifications already at the present stage of knowledge. Previous estimates predicted that half of the population at highest risk may account for about 88% of breast cancer cases [94]. Using the current set of loci and assuming that all loci combine multiplicatively, risks of breast cancer were estimated approximately 2 · 3-fold and 3-fold higher for individuals in the top 5% and 1% of the population, relative to the population average [72]. With the identification of many more low-risk loci it may become possible to calculate combinatorial risks that could be useful in a stratified approach of cancer prevention in the future [148-150].

Population diversity needs to be taken into account for breast cancer susceptibility at all levels of penetrance. Due to founder effects, single mutations can contribute significantly to the breast cancer burden in founder populations and intermediate-risk alleles in some genes have almost exclusively been found in certain population groups, such as for *FAM175A* and *RAD50* in the Finnish population or *NBN* in Slavic populations [28,41-44]. In fact, much of the present knowledge about those genes relies on particular founder mutations, and in regard of allelic heterogeneity one must be cautious to extrapolate and generalise these observations to other less common alleles. Similarly, common polymorphisms at breast cancer susceptibility loci will differentially impact on breast cancer risk in different ethnic groups, if they display different frequencies or different linkage disequilibrium patterns across populations, such as CASP8*D302H that is virtually absent in Asians [121], or the *ESR1* locus at which different risk alleles SNPs have emerged in Asians and Europeans [100,103,125-127]. Gene-based strategies for an improved risk prediction will therefore need to be elaborated in a population-specific way.

In addition to risk prediction, identifying the genetic basis of breast cancer in the individual patient might have further prognostic and therapeutic implications. Breast cancer therapy has been guided for long by the presence or absence of gene products such as hormone receptors or HER2/neu. These tumour characteristics are partly determined by germ-line mutations, as exemplified by *BRCA1* mutations which are frequently associated with triple-negative breast cancers, but breast cancer pathology also seems to be influenced by low-penetrance variants like those in *FGFR2* that are strongly correlated with estrogen-receptor positive disease [95,96,151]. In fact, many of the hitherto identified variants appear to preferentially associate with a defined estrogen receptor status (Table 2) [119,123,152]. Further studies are presently underway to investigate whether SNP profiling could be of prognostic value [153].

The identification of breast cancer susceptibility alleles may also guide the development of new drugs that target additional breast cancer pathways, such as oncogenic signalling mediated by FGF receptors [154] or mutation accumulation mediated through ABOBEC3B. Such new drugs are particularly needed in the treatment of otherwise poorly targetable breast carcinomas such as triple-negative tumours [155] and the identification of risk alleles in genes like *BABAM1* or *MDM4* in this particular subgroup may offer promising avenues for new therapeutic regimens. The concept of “synthetic lethality” as exemplified by the introduction of PARP1 inhibitors into treatment of patients with *BRCA1* or *BRCA2* mutations may also prove useful in the development of other compounds to target additional genetic predispositions [156-158].

## Conclusions and outlook

Tremendous progress has been made during the past few years in deciphering the polygenic susceptibility to breast cancer. The results suggest that key pathways are targeted by different sources of genetic variation influencing the hereditary risk. To a large extent these findings fulfil the predictions made some forty years ago that “genes may either cause susceptibility of the mammary gland to hormonal action [or to a virus], or induce an easy transformation from a normal to a malignant cell” [1]. It can be anticipated that hundreds of additional loci are still to be detected that collectively form the basic layout for an individual’s susceptibility to breast cancer. With many more genes being identified, a deeper understanding of breast cancer development and progression together with the ability of gene-based stratification should ultimately lead to improved prevention and an individually tailored therapy to the benefit of each patient.

## Abbreviations

CNV: Copy number variation; GWAS: Genome-wide association study; SNP: Single nucleotide polymorphism; TNBC: Triple-negative breast cancer.

## Competing interests

The authors declare that they have no competing interests.

## Authors’ contributions

NB worked on Table 1 and Figure 1. SH worked on Table 2 and Figure 2. TD drafted the manuscript. All authors read and approved the final manuscript.

## Helpful websites

NCBI PubMed http://www.ncbi.nlm.nih.gov/pubmed

NCBI Genbank http://www.ncbi.nlm.nih.gov/gene

NCBI SNP database http://www.ncbi.nlm.nih.gov/snp

NIH GWAS Catalogue http://www.genome.gov/gwastudies/

iCOGS information http://www.nature.com/icogs/ , http://www.nature.com/ng/journal/v45/n4/full/ng.2592.html

## Search strategy and selection criteria

References for this Review were identified through searches of PubMed with the search terms “breast cancer”, “mutation”, and “genetic susceptibility” from 1990 until March, 2013. Articles were also identified through searches of the authors’ own files. Only papers published in English were reviewed. The final reference list was generated on the basis of originality and relevance to the broad scope of this Review. We apologize to all authors whose work may have been undeliberately overlooked using this approach.
